# Association between Physiological and Subjective Aspects of Pain and Disability in Post-Stroke Patients with Shoulder Pain: A Cross-Sectional Study

**DOI:** 10.3390/jcm8081093

**Published:** 2019-07-24

**Authors:** Lydia Martín-Martín, Miguel David Membrilla-Mesa, Mario Lozano-Lozano, Noelia Galiano-Castillo, Carolina Fernández-Lao, Manuel Arroyo-Morales

**Affiliations:** 1 Department of Physical Therapy, Instituto Biosanitario Granada (IBS.Granada), University of Granada, 18071 Granada, Spain; 2 Physical Medicine and Rehabilitation Department, Section Rehabilitation and Traumatology, Hospital Virgen de las Nieves, 18014 Granada, Spain; 3 Department of Physical Therapy, Instituto Biosanitario Granada (IBS.Granada), Instituto Mixto Deporte y Salud (iMUDS), University of Granada, 18071 Granada, Spain

**Keywords:** stroke, pain, disability, central sensitization, pressure pain thresholds

## Abstract

*Background*: Patients often experience pain as a result of a stroke. However, the mechanism of this pain remains uncertain. Our aim was to investigate the relationship between pressure pain thresholds (PPTs) and disability pain in patients with hemiplegic shoulder pain (HSP). *Methods*: Twenty-six post-stroke patients (age 53.35 ± 13.09 years) and healthy controls (54.35 ± 12.37 years) participated. We investigated spontaneous shoulder pain, disability pain perception through the shoulder pain and disability index (SPADI), and the PPTs over joint C5–C6, upper trapezius, deltoid, epicondyle, second metacarpal, and tibialis anterior, bilaterally. *Results*: The analysis of variance (ANOVA) showed significant differences in pain between groups (*p* < 0.001) and differences in the SPADI (*p* < 0.001) between groups but not between sides for PPTs over deltoid (group: *p* = 0.007; side: *p* = 0.750), epicondyle (group: *p* = 0.001; side: *p* = 0.848), and tibialis anterior (group: *p* < 0.001; side: *p* = 0.932). Pain in the affected arm was negatively associated with PPTs over the affected epicondyle (*p* = 0.003) and affected tibialis anterior (*p* = 0.009). Pain (SPADI) appeared negatively correlated with PPTs over the affected epicondyle (*p* = 0.047), and disability (SPADI) was negatively associated with PPTs over the affected tibialis anterior (*p* = 0.041). *Conclusions*: Post-stroke patients showed a relationship between widespread pressure pain hypersensitivity with lower PPT levels and pain disability perception, suggesting a central sensitization mediated by bilateral and symmetric pain patterns.

## 1. Introduction

Stroke or cerebrovascular disease is considered a highly prevalent condition, which is one of the leading causes of chronic disability and dementia [1]. In Spain, the estimated annual incidence is 118 per 100,000 people, and it is the principal cause of death among Spanish women [2]. Strokes are the leading cause of long-term disability, associated with various consequences [3] including impaired upper limb motor function [4], physio-emotional stress, and socioeconomic problems for the patients, their families, and health systems [5]. 

It is very common to experience pain as a result of a stroke, and it can be described as either neuropathic pain (central post-stroke pain) or nociceptive pain (shoulder and other musculoskeletal pains) [6]. A possible explanation of the underlying pathophysiologic mechanisms could be based on the clinical characteristics of the condition, such as sensory loss, hypersensitivity, and decreased or increased sensations of temperature and/or pain [6]. In fact, an injury in the central nervous system can produce anatomical, neurochemical, excitotoxic, and inflammatory problems that could induce and increase neuronal excitability [7]. Specifically, hemiplegic shoulder pain (HSP) has an incidence of 21–72% according to some studies [8,9], and this pain is often accompanied by pain in additional areas on the upper limb [8]. The research has shown that this shoulder pain is associated with interference of daily activities and a reduced quality of life [10]. 

The mechanism of post-stroke pain remains uncertain and is considered multifactorial and complex [11,12]. The significance of musculoskeletal factors in the etiology of post-stroke pain is also unclear, although different studies have analyzed muscle pain through pressure pain thresholds (PPTs) within this population [13,14]. PPTs and spontaneous pain are significant and common parameters in the research of muscle pain in different conditions [15]. PPT is very useful at measuring chronic pain in the upper limb and chest [16] and provides a quantitative assessment of sensory perception of mechanical stimuli. An increase in pain perception in tissues near to the site of damage as well as tissues at a distant area have been shown in central sensitization [17], and a method of measuring central sensitization is measuring mechanical hyperalgesia through PPTs [13].

Pain thresholds are influenced by several factors in addition to the disease process, and this must be taken into account [18,19]. Unidimensional pain rating scales alone reflect little information on the somatosensory nature of pain, so they reveal more specifically the emotional status of the patient. Perceived pain is considered multifactorial, and the emotional and the physical status are important aspects of this pain. In this respect, some previous reports have stated the association of these different aspects of pain by assessing various populations [20,21], and they have studied these aspects and their relationship in people with neck pain [18]. All of the results revealed a negative association; the lower the PPTs, the higher the perceived pain.

To our knowledge, the relationship between physiological parameters of pain and aspects based on a more objective assessment in post-stroke patients with HSP are not extensively understood. As a result, it may be considered beneficial to evaluate the relationship between self-reported intensity of pain methods (unidimensional and multidimensional) and quantitative reported pain methods.

The aim of this study was to investigate the relationship between pressure pain thresholds, disability, and self-perception of pain in a population of post-stroke patients with HSP. 

## 2. Experimental Section

### 2.1. Study Design

We conducted an observational cross-sectional study.

### 2.2. Participants

Post-stroke patients with HSP were recruited from the Physical Medicine and Rehabilitation Service in the Virgen de las Nieves University Hospital, Granada, Spain, from March 2016 to April 2017. The inclusion criteria were: (a) subjects >18 years, (b) unilateral ischemic or hemorrhagic stroke, (c) pain in the shoulder area during rest or during passive joint mobilization, (d) post-stroke pain or pain exacerbated by stroke according to medical criteria based on advanced images of the affected shoulder, and (e) shoulder pain for six months or more. The exclusion criteria were: (a) previous shoulder surgery, (b) presence of another chronic pain syndrome (i.e., previous stroke and hemiplegia), and (c) cognitive or communicative impairments such as hemineglect or aphasia that may impede assessment. 

Additionally, age- and gender-matched controls were recruited from volunteers who responded to a local announcement about the study, and candidates were excluded if they exhibited a history of neck, shoulder, or arm pain, history of trauma, or diagnosis of any systemic disease. The study protocol was approved by the Biohealth Ethics Committee of the province of Granada (RHB 02) and conducted following the Helsinki Declaration. All participants signed an informed consent form prior to their inclusion in the study.

All assessments and tests were administered in the morning, always with at least one hour before or after finishing a meal. 

### 2.3. Outcome Measures

#### 2.3.1. Pain Intensity (Numerical Point Rating Scale—NPRS)

We used an 11-point numerical point rating scale (NPRS) [22] (where 0 = no pain; 10 = maximum pain) to assess the intensity of spontaneous HSP. The patients were required not to take analgesics or muscle relaxants for 24 h prior to the assessment.

#### 2.3.2. Shoulder Pain and Disability Index (SPADI)

The shoulder pain and disability index (SPADI) is a 13-item questionnaire designed to measure current shoulder pain and disability in an outpatient setting. The SPADI assessment uses two scales: a 5-item subscale that measures pain and an 8-item subscale that measures disability. The reliability coefficients of the intraclass correlation coefficient (ICC) average is 0.89 (95% CI 0.66–0.95) in a variety of patient populations [23], and the Spanish version [24] demonstrated satisfactory psychometric properties in the population with different shoulder disorders. 

#### 2.3.3. Pressure Pain Thresholds (PPTs)

PPTs were assessed bilaterally over different points in the neck, shoulder, and arm areas (zygapophyseal joints C5–C6, middle point of the upper trapezius muscle, deltoid muscle, epicondyle, second metacarpal, and tibialis anterior muscle as a distant site). We used a Force Dial FDK 20 with a steel probe covered by a rubber surface with a 1.0 cm^2^ area (Wagner Instruments, Greenwich, CT). Pressure was applied gradually at 1 kg/s. The subjects were required to assume laying position, made comfortable, and encouraged to maintain complete relaxation. The subjects were then instructed to indicate when the sensation first changed from pressure to pain. The mean of three trials was then calculated and used for the main analysis, and a 20 s resting period was maintained between each trial. Reliability of the results of this procedure has been found to be high when recorded during the same day (ICC) = 0.91 (95% CI 0.82–0.97)) [25].

### 2.4. Sample Size Calculation

Sample size calculation and power determination were implemented using software (EPIDAT 3.1, Xunta de Galicia, Spain). The calculation of sample size was carried out by detecting, at least, significant clinical differences of 20% based on a previous study [26] on the cervical area, on PPT levels between both groups with an alpha level of 0.05, a desired power of 80%, and an estimated inter-individual coefficient of variation of PPT measures of 20%. This procedure generated a sample size of at least 16 subjects per group. Finally, we included 26 participants to allow for potential dropouts. 

### 2.5. Statistical Analysis

Data results were analyzed with the SPSS statistical package (version 22.0). Results are expressed as mean, standard deviation (SD), or 95% confidence interval (CI). We used the Kolmogorov–Smirnov test to analyze the normal distribution of the variables (*p* > 0.05). Differences in the results for shoulder pain intensity (NPRS) and the SPADI questionnaires were assessed for both groups with the Mann–Whitney *U* test. A two-way analysis of variance (ANOVA) was used to investigate the differences in PPT assessed over each point with side (affected/nonaffected within patients or dominant/nondominant controls) as within-subject factor and group (patients with HSP or healthy controls) as between-subject factor. Finally, an analysis of correlation was conducted to evaluate the association between subjective and physiological variables. The statistical analysis was conducted at 95% confidence level. A *p* < 0.05 was considered statistically significant. An analysis of covariance (ANCOVA) was used to identify group differences on the outcome variables, controlling for age.

## 3. Results

### 3.1. Demographic and Clinical Data

The participants of the study were 26 post-stroke patients aged 20–77 years old (mean 53.35 ± 13.09), 15 of which were men (57.7%), and 23 healthy controls aged 25–69 years old (mean 54.35 ± 12.37), 14 of which were men (60.9%). No statistical differences between sex were found (*p* = 0.821).

Within the patient group, 23 (88.5%) had spastic muscle tone and 3 (11.5%) had flaccid tone; 15 (57.7%) had hemiplegia on their left side and 11 (42.3%) had hemiplegia on their right; and 25 (96.2%) were right-handed, whereas the remaining were left-handed. The average time from event was 82.38 ± 64.43 months. Among all patients, 21 (80.8%) had received botulinum toxin as treatment for muscle tone exacerbation.

### 3.2. Pain Intensity (NPRS)

The patient group reported moderate levels of perceived pain in their affected arm (mean intensity: 5.01 ± 3.63), and the healthy control group showed no pain in their dominant arm (mean intensity: 0.09 ± 0.42) (*p* < 0.001). The patients exhibited almost no pain in their nonaffected arm (mean intensity: 0.58 ± 1.69), and the healthy group exhibited no pain at all in their nondominant arm (mean intensity: 0) (*p* = 0.021) (Table 1). The ANCOVA analysis of pain intensity by age showed no significant differences, neither for the affected arm nor for the nonaffected (*f* < 0.001, *p* = 0.983)

### 3.3. Shoulder Pain and Disability Index (SPADI)

Regarding shoulder pain and disability, the group of people with hemiplegia perceived higher levels of pain and disability (mean 36.27 ± 32.10 and 93.25 ± 13.11, respectively) than the control group (mean 1.70 ± 6.06 and 0.12 ± 0.52, respectively), and they had higher levels of the total score (mean 73.42 ± 13.83 and 0.72 ± 2.47, respectively) (*p* < 0.001 for all) (Table 1). The ANCOVA analysis of shoulder pain and disability by age showed no significant differences for perceived pain (*f* = 0.160, *p* = 0.691), for perceived disability (*f* = 1.392, *p* = 0.244), nor for the total score (*f* = 1.779, *p* = 0.189).

### 3.4. Pressure Pain Thresholds (PPTs)

**Figure 1:**
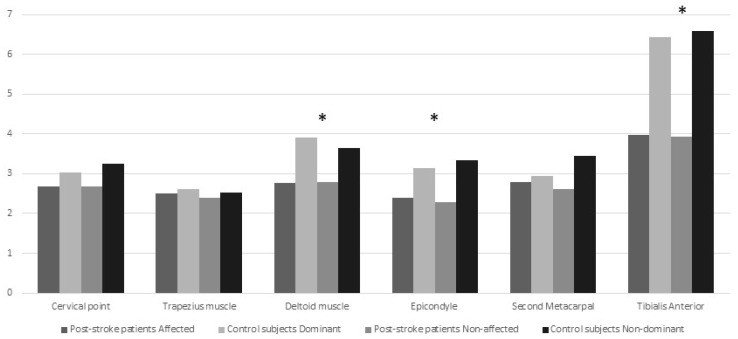
Representation of pressure pain threshold levels (kg/cm^2^) in post-stroke patients and healthy controls on each point. * Significant differences between post-stroke patients and control subjects.

The ANOVA revealed significant differences between patients and controls but not between sides for PPT levels over the deltoid muscle (group: *f* = 7.527, *p* = 0.007; side: *f* = 0.103, *p* = 0.750), the epicondyle (group: *f* = 12.791, *p* = 0.001; side: *f* = 0.037, *p* = 0.848) and tibialis anterior muscle (group: *f* = 25.547, *p* < 0.001; side: *f* = 0.007, *p* = 0.932). No significant interactions between group × side were found: cervical point (*f* = 0.168; *p* = 0.683), trapezius muscle (*f* < 0.001; *p* = 0.985), deltoid muscle (*f* = 0.162; *p* = 0.689), epicondyle (*f* = 0.373; *p* = 0.543), second metacarpal (*f* = 0.326; *p* = 0.569), and tibialis anterior (*f* = 0.034; *p* = 0.854). Table 2 and Figure 1 summarizes PPT levels assessed over the muscles for both sides within each study group. The ANCOVA analysis of pressure pain thresholds by age showed similar figures: significant differences between patients and controls but not between sides for PPT levels over the deltoid muscle (group: *f* = 7.186, *p* = 0.009; side: *f* = 0.091, *p* = 0.763), the epicondyle (group: *f* = 12.369, *p* = 0.001; side: *f* = 0.043, *p* = 0.836), and tibialis anterior muscle (group: *f* = 25.850, *p* < 0.001; side: *f* = 0.005, *p* = 0.943). No significant interaction between group × side was found: cervical point (*f* = 0.165; *p* = 0.68), trapezius muscle (*f* = 0.001; *p* = 0.980), deltoid muscle (*f* = 0.178; *p* = 0.674), epicondyle (*f* = 0.354; *p* = 0.553), second metacarpal (*f* = 0.327; *p* = 0.569), and tibialis anterior (*f* = 0.039; *p* = 0.844).

### 3.5. Correlations of PPT with NPRS and SPADI

In the post-stroke patient group, perceived pain (NPRS) in the affected arm was negatively associated with PPT levels over the affected epicondyle (*r* = −0.453; *p* = 0.003) and affected tibialis anterior point (*r* = −0.404; *p* = 0.009). In summary, the greater the pain in the affected arm, the lower the PPT levels reported. 

Pain in the SPADI showed negative correlation with PPT levels over the affected epicondyle (*r* = 0313; *p* = 0.047). Meanwhile, disability (SPADI) was also negatively associated with PPT level over the affected tibialis anterior point (*r* = −0.325; *p* = 0.041). Namely, the lower the disability, the greater PPT level on that point.

## 4. Discussion

The main objective of the study was to analyze the muscular PPTs in post-stroke patients with HSP, the relationship between them, and the disability and pain perception in this population. The results showed a widespread, bilateral hypersensitivity in post-stroke patients, associated with central sensitization, when compared with the control group. Besides that, our work shows an association between the PPT values through an algometry assessment as well as the disability and pain perception through the NPSRS and SPADI questionnaire. This association demonstrates that patients with lower PPT levels report more severe arm symptoms. 

Patients with chronic HSP showed a bilateral, widespread hypersensitivity objectified by significant lower PPT levels over deltoid, epicondyle, and anterior tibial muscle points compared to healthy matched controls. A recent study has pointed out that in central post-stroke pain, the afferent sensory input from the painful area plays a role in maintaining the spontaneous and the evoked pain [27], and many different previous works have shown a possible central sensitization in this population [13,14,28]. This central sensitization may modify the normal processing of pain stimuli that generate hypersensitivity by neuroplasticity [29]. In fact, a recent work [30] has suggested that the HSP is associated with a poor pain adaptation in the painful shoulder and in the nonaffected side of the patient. In post-stroke patients, spontaneous muscle pain in the hemiplegic side is common [14], but surprisingly, in our study, the PPT levels were slightly lower within the unaffected side than the affected side, although these were not significantly different. Perhaps this fact could be explained by a more important altered superficial sensibility [31] on the hemiplegic side in these patients. However, within the rest of the points assessed in our study, the PPT levels were lower in both sides when compared with healthy controls, but these differences were not statistically significant. It is possible that the points assessed were related to the neck area, and this region is more sensitive in the general population around the mean age studied [18]. 

This phenomenon might be related to a so-called central or sensitization mechanism. Previous works have found central sensitization in other pain disorders such as carpal tunnel syndrome and shoulder impingement syndrome [20,21], where chronic pain in the shoulder may be related to lower PPT levels in the lower leg muscle. This fact might explain why central post-stroke pain or other pain syndromes could also involve the healthy side of stroke patients [32]. Sensitized pressure pain hyperalgesia might be sustained by peripheral noxious inputs, which are input from post-stroke hemiparetic pain into the central nervous system, but other mechanisms such as brain injury coming from the stroke itself or an abnormality in muscle balance could also contribute.

Correlation analysis showed a positive association between pressure pain thresholds, disability, and pain perception, demonstrating that disability increased proportionately to the intensity of muscular pain. There are a growing number of papers that highlight the relation between subjective symptom perceptions of patients with different conditions and the results obtained in different objective physical evaluation methods. Lindgren et al. [33] found an association between shoulder pain perception, assessed by a visual analogue scale, and upper extremity sensorimotor function, assessed by passive mobilizations or light touch, demonstrating the correlation between the results of a pain self-reported measure and objective assessments in post-stroke patients. Another earlier work [18] showed correlations between PPTs and NPRS scores in people with neck pain, and an inversely proportionate relation between PPTs and disability was also found in patients with chronic spinal pain by Moura et al. [34]. Fernández de las Peñas et al. [20] also discovered a correlation between pain intensity and bilateral PPTs in patients with carpal tunnel syndrome. Finally, a recent paper [35] described an association of PPTs over the masseter muscle and temporomandibular reported disorders. In contrast to this, Kamper et al. [36] found a weak association between intensity of reported neck pain and PPTs in people after whiplash. There is also previous research that did not find any correlation between pressure pain thresholds and reported pain intensity in people with temporomandibular disorders [37]. Specifically, we found an association between disability in the SPADI, but not with the pain, and the PPT level over the affected tibialis anterior muscle. It could be suggested that post-stroke patients more strongly relate disability with impaired mobility and spasticity in the affected leg than with the pain perceived. 

Although, to our knowledge, this is the first study investigating the association between pressure pain thresholds, disability, and pain perception in post-stroke patients with central sensitization, some limitations should be taken into account. One of the more significant limitations is the absence of a group of post-stroke patients without pain in our study. Further research including different groups of post-stroke patients with different characteristics may strengthen the results. However, correlation between the physiological and subjective variables of pain and disability highlight the necessity of evaluating these types of parameters together to assess the chronic pain in post-stroke populations. Besides that, the addition of a longer follow-up period would provide more information about the evolution of sensitization processes in post-stroke patients. A larger sample size would be required to permit a more general interpretation of the results. Finally, this is a single-center study focusing only on one specific hospital and population served, so the results cannot be extrapolated. 

## 5. Conclusions

This study found a relationship between the presence of widespread pressure pain hypersensitivity in post-stroke patients with HSP, objectified by lower PPT levels, and pain disability perception evaluated by questionnaires, suggesting a central sensitization, mediated by bilateral and symmetric pain patterns.

## Figures and Tables

**Table 1:** Pain and disability perception in post-stroke patients and healthy controls.

	Post-Stroke Patients (*n* = 26)	Control Subjects (*n* = 23)
**NPRS**		
Affected/dominant	5.01 ± 3.63 (3.85–6.17) *	0.09 ± 0.42 (−0.93–0.27)
Nonaffected/nondominant	0.58 ± 1.69 (0.03–1.12) *	0
**SPADI**		
Pain	36.27 ± 32.10 (26.00–46.53) *	1.70 ± 6.06 (−0.92–4.31)
Disability	93.25 ± 13.11 (89.06–97.44) *	0.12 ± 0.52 (−0.12–0.33)
Total	73.42 ± 13.83 (69.00–77.85) *	0.72 ± 2.47 (−0.35–1.79)

Values are mean ± SD (95% confidence interval). * Significant differences between post-stroke patients and control subjects (analysis of variance test). NPRS, numerical pain rating scale. SPADI, shoulder pain and disability index.

**Table 2:** Pressure pain thresholds (kg/cm^2^) in post-stroke patients and healthy controls.

	Cervical Point	Trapezius Muscle	Deltoid Muscle *	Epicondyle *	Second Metacarpal	Tibialis Anterior *
**Post-stroke patients**						
Affected	2.69 ± 1.71 (1.74–3.60)	2.50 ± 1.98 (1.54–3.27)	2.77 ± 1.77 (1.92–3.67)	2.39 ± 1.16 (1.75–2.82)	2.78 ± 1.94 (1.97–3.27)	3.98 ± 2.00 (2.95–4.90)
Nonaffected	2.67 ± 2.04 (2.00–3.39)	2.40 ± 1.90 (1.70–3.30)	2.80 ± 1.92 (2.05–3.48)	2.28 ± 1.18 (1.92–2.86)	2.62 ± 1.43 (2.00–3.56)	3.92 ± 2.15 (3.17–4.79)
**Control subjects**						
Dominant	3.02 ± 1.07 (2.84–3.67)	2.62 ± 0.56 (2.11–2.92)	3.91 ± 1.56 (2.90–4.40)	3.13 ± 1.17 (2.76–3.91)	2.95 ± 0.82 (2.60–3.31)	6.44 ± 2.38 (5.25–7.92)
Nondominant	3.25 ± 0.97 (2.56–3.49)	2.52 ± 0.94 (2.38–2.87)	3.65 ± 1.74 (3.23–4.59)	3.34 ± 1.32 (2.63–3.64)	3.45 ± 1.10 (2.97–3.92)	6.58 ± 3.09 (5.42–7.47)

Values are mean ± SD (95% confidence interval). * Significant differences between post-stroke patients and control subjects (two-way analysis of variance test).
